# Immunoinflammatory cells and Beta-defensin-3 in radicular cysts of primary and permanent teeth

**DOI:** 10.1590/1807-3107bor-2026.vol40.024

**Published:** 2026-05-18

**Authors:** Amanda Silva Bertasso, Jorge Esquiche Léon, Olívia Santana Jorge, Raquel Assed Bezerra da Silva, Marília Pacífico Lucisano, Alexandra Mussolino de Queiroz, Evânio Vilela da Silva, Léa Assed Bezerra da Silva, Paulo Nelson-Filho

**Affiliations:** (a) Universidade de São Paulo – USP, School of Dentistry of Ribeirão Preto, Department of Pediatric Dentistry, Ribeirão Preto, SP, Brazil.; (b) Universidade de São Paulo – USP, School of Dentistry of Ribeirão Preto, Department of Stomatology, Public Oral Health and Forensic Dentistry, Ribeirão Preto, SP, Brazil.

**Keywords:** Immunohistochemistry, Radicular Cyst, Tooth, Deciduous, Beta-Defensins

## Abstract

The expression of hBD-3 and quantification of immune and inflammatory response cells (non-degranulated and degranulated mast cells, mature and immature plasmacytoid dendritic cells, mature and immature Tregs, T lymphocytes, cytotoxic T lymphocytes, and B lymphocytes) were evaluated in the radicular cysts (epithelium/capsule) of primary and permanent teeth. The relationship between the size of the radiographic lesion and expression of hBD-3 was also evaluated. Radicular cysts were subjected to immunohistochemical analysis to quantify the immune and inflammatory response cells and to evaluate hBD-3 staining and its relationship with radiographic lesion size. The results were analyzed using the D’Agostino & Pearson, Mann–Whitney, t-test, Kruskal–Wallis, and Dunn's post-tests (5%). hBD-3 was expressed in cysts of primary and permanent teeth. In primary teeth, hBD-3 expression was higher in small lesions than in large lesions (p < 0.05). All the evaluated cell types were detected in all radicular cysts. Cysts of primary teeth showed a higher expression of plasmacytoid dendritic cells, B lymphocytes, and T lymphocytes (p < 0.05), whereas those of permanent teeth showed a higher expression of T lymphocytes, immature plasmacytoid dendritic cells, cytotoxic T lymphocytes, and B lymphocytes (p < 0.05). hBD-3 was expressed in the epithelium/capsule of primary and permanent teeth radicular cysts. Immature plasmacytoid dendritic cells were the predominant cells in radicular cysts of primary teeth, whereas T lymphocytes were more abundant in permanent teeth.

## Introduction

Oral infectious diseases, such as dental caries, periodontitis, and apical periodontitis, are often complicated by causative bacterial biofilm formation and significantly impact human oral health and quality of life.^1^ Periapical injury represents an inflammatory condition of the periradicular tissues due to microbial colonization of the root canal system.^2^ When the periapical region is invaded by microorganisms, their products, or byproducts, the innate immune system is activated and is associated with the migration of mast cells, neutrophils, macrophages, and dendritic cells, among other cells, to the lesion site. Subsequently, cells of the adaptive immune system, including T and B lymphocytes, migrate to that site.^3^

The role of the immune system in periapical lesions has been previously evaluated. Macrophages,^4,5^ regulatory T lymphocytes (Tregs),^6^ cytotoxic T lymphocytes,^4^ mast cells^7–9^ and dendritic cells,^10^ among others, are present in the periapical lesions of permanent teeth. However, the literature in this regard for the primary dentition is scarce.^3,11,12^ it is known that in the first years of a child's life, the immune system is immature, which makes them vulnerable to infectious diseases.^13^ Due to the lack of studies characterizing and quantifying the different cell types of the immune response in primary teeth with pulp necrosis and periapical lesions and considering the potential systemic effects of infection and the reactions triggered in children,^13^ it is relevant to carry out studies in this regard. Furthermore, this knowledge can provide a better understanding of pulp and periapical pathology in primary teeth than in permanent teeth, favoring the development and use of more targeted treatment techniques.^14–16^

Besides cell-mediated innate immunity against microorganisms, small cationic or anionic peptides, known as antimicrobial peptides (AMPs), exhibiting antimicrobial activity, are also a part of innate immunity.^17^ Beta-defensins are important AMPs^18^ produced by epithelial cells in response to microbial infections in pathologies such as mucoceles,^19^ dental caries,^20^ periodontal disease,^21^ asthma,^22^ colitis^23^ and psoriatic lesions,^24^ among others. It is also considered as a functional biomarker of oral cancer.^25^ However, studies on beta-defensins and endodontics are scarce and have mostly been restricted to *in vitro* experiments on permanent teeth^26–31^ or to an animal model.^32,33^ To the best of our knowledge, no studies have evaluated whether beta-defensin is produced in the cysts of human primary and permanent teeth.

Thus, it is concluded that beta-defensins play multiple roles in the human body, including broad-spectrum antimicrobial activity, influence on the repair process, angiogenesis^35^ and reduction in the production of pro-inflammatory cytokines stimulated by Lipopolysaccharides (LPS).^36^ The specific literature has demonstrated that hBD-3 exhibits both antimicrobial and immunomodulatory activities.^37^ Thus, its can be hypothesized that during the development of periapical lesions, smaller lesions could result, among other factors, from a more effective production of these antimicrobial peptides as a response of the organism to microbial infection of the root canals and this response could be different in primary teeth and in permanent teeth.

Cell- and beta-defensin-mediated immunity provides the main defense against the installation and progression of periapical lesions, especially in radicular cysts. Therefore, clinical studies are needed to evaluate immune and inflammatory responses, as well as to identify endogenous AMPs, for a better understanding of pulpal and periapical pathologies, particularly in primary teeth, and, as pointed out by Morio et al.,^38^ for discovering possible therapeutic targets; for example, by inducing the expression of endogenous defensins.

In the above-mentioned context, this study aimed to quantify hBD-3 expression, inflammatory and immune cells, namely mast cells (non-degranulated and degranulated), plasmacytoid dendritic cells (mature and immature), Tregs (mature and immature), T lymphocytes, cytotoxic T lymphocytes, and B lymphocytes, and in radicular cysts of primary and permanent teeth using immunohistochemistry. Additionally, the relationship between radiographic lesion size and hBD-3 staining intensity was evaluated.

## Methods

This study was approved by the ethics committee of the Faculty of Dentistry of Ribeirão Preto, University of São Paulo (protocol number 80253417.6.0000.5419). All the patients received a consent form and after agreeing, they signed authorizing participation.

### Sample selection

The sample size calculation for assessment of hBD-3 expression was based on the results of Frederic et al.^21^ The sample power was calculated considering the mean and standard deviation of the evaluated groups, obtaining a β error of 0.80 and test power of 84%, which established the need for at least 9 lesions for each group.

For the assessment of inflammatory cells, the sample size calculation was based on the results of Bertasso et al.^12^ The sample power was calculated by considering the mean and standard deviation of the evaluated groups, obtaining a β error of 0.8 and test power of 81%, which established that at least 16 lesions for each group would be adequate for a scientifically valid result.

### Histotechnical processing

Following the medical history assessment, the patients included in the study were required to be in good general health and not have used systemic medications within the preceding three months. Patients with teeth with indications for extraction, absence of pain, presence/absence of fistulae, extensive coronal destruction due to caries lesions without any possibility of restoration, pulp necrosis, and radiographically visible apical periodontitis and no previous treatment were included.

During the extraction, the periapical lesion was removed and fixed in 10% buffered formalin for 24 h, washed for 24 h, dehydrated in alcohol, cleared in xylene, and embedded in paraffin. From the paraffin blocks, 10 consecutive 3 μm thick sections were obtained and mounted on glass slides. One section was stained with hematoxylin and eosin (HE) for histopathological diagnosis of the lesions. The remaining sections were used for immunohistochemical analysis.^12^

Nineteen primary and 17 permanent teeth with a histopathological diagnosis of radicular cysts in the hematoxylin and eosin-stained sections were selected.

### Immunohistochemical analysis

Sequential 3 μm thick sections of each specimen were mounted on silanized slides for immunohistochemical analysis using the avidin-biotin-peroxidase complex method.^12^

The sections were deparaffinized and hydrated as previously described. Antigenic retrieval was performed by immersing the slides in citrate buffer (pH = 6.0) and heating them in a microwave oven at maximum power (2 cycles of 10 s each). After cooling to room temperature (23°C), the slides were washed twice (10 min each time) with phosphate-buffered saline (PBS) and rinsed for 10 min with 0.5% PBS/Triton solution (Sigma-Aldrich Corp, St Louis, USA). Endogenous peroxidase was blocked by immersing the slides in a 3% hydrogen peroxide solution for 20 min in the dark, followed by rinsing in PBS and PBS/Triton solution as described previously.^12^ Nonspecific binding was blocked by immersing the slides in a 1% bovine serum albumin (BSA)/PBS solution for 30 min. The slides were subsequently incubated overnight at 4°C with the primary antibodies diluted in 1% BSA. Thereafter, the slides were brought to room temperature, washed, and incubated for 30 min with biotinylated secondary antibody (goat antirabbit immunoglobulin G-B and rabbit antirabbit immunoglobulin G-B, Santa Cruz Biotechnology Inc.; 1:200 dilution) for 1 h at room temperature. After washing with PBS and PBS/Triton solution, the slides were incubated with a streptavidin-biotin-peroxidase complex (ABC Kit, Vecstain; Vector Laboratories Inc., Burlingame, USA). The slides were washed again with PBS and PBS/Triton solution, and the reaction was visualized using the chromogen, 3,3’ diaminobenzidine tetrahydrochloride hydrate (DAB; Sigma-Aldrich Corp, St Louis, USA) mixed with 3% hydrogen peroxide in PBS for 1 min. The slides were counterstained with Harris hematoxylin for 10 s, washed in running water and ammoniacal water for 30 s, and then again in running water. The sections were cleared, dehydrated, and covered with coverslips. Table 1 lists the specifications of primary antibodies used for cell identification (Table 1).

**Table 1:** Specifications of the primary antibodies used in the study

Biomarker		Clone	Description	Isotype	Dilution	Brand	Cell	Positive control
Tryptase		AA1	Monoclonal mouse	IgG1	3,51388889	DakoCytomation	Mast cell	Tonsils
CD303		124B3.13	Monoclonal mouse	IgG1	0,38888889	Dendritics	Dendritic cell	Tonsils
CD123		BR4MS	Monoclonal mouse	IgG2b	0,18055556	Leica Biosystems	Dendritic cell	Tonsils
CD25		B1.49.9	Monoclonal mouse	IgG2a	0,31944444	Beckman	Regulatory T cell	Tonsils
FOXP3		SP97	Monoclonal rabbit	IgG	0,25	Spring Bioscience	Regulatory T cell	Tonsils
	CD3	A0452	Polyclonal rabbit	IGg1	0,38888889	DakoCytomation	T Lymphocyte	Tonsils
	CD8	C8/144B	Monoclonal mouse	IGg	0,31944444	DakoCytomation	T Lymphocyte	Tonsils
	CD20	L26	Monoclonal mouse	IgG	1,43055556	DakoCytomation	B Lymphocyte	Tonsils
hBD-3		23-67/67	Polyclonal	IgG	0,31944444	Bioss	h-BD3	Lung, colon e tonsils

Quantification of immune and inflammatory response cells. The slides were analyzed as described by Bertasso et al.^12^ using an Axio Imager. An M1 (Carl Zeiss Microimaging GmbH, Gottingen, Germany) microscope equipped with a camera and a previously calibrated examiner (Kappa > 0.9) was used at ×10, ×20, and ×400 magnifications. The ×10 magnification was used to select the regions with the most intense immunostaining, and ×400 magnification was used to obtain images from five representative regions in each specimen. After image acquisition, immunostaining was assessed using Image J 1.28 software (National Institute of Health, Bethesda, USA).

Initially, each slide was evaluated for the presence or absence of each cell type (mast cells (non-degranulated and degranulated), plasmacytoid dendritic cells, cytotoxic T lymphocytes, Tregs, T lymphocytes, and B lymphocytes), and the number of positive cells in each field was counted and averaged (quantitative analysis). The results were expressed as the number of cells of each type per specimen.

### Quantification of the antimicrobial peptide (hBD-3)

From the initial cases, immunohistochemical analysis of hBD-3 was performed on 14 cases of primary teeth and 13 cases of permanent teeth. For each slide, the presence or absence of hBD-3 immunostaining was analyzed separately at two different sites: the cystic capsule and the cystic epithelium. Semi-quantitative analysis of immunostaining at these two sites was performed according to the following criteria:^33^

- Score 0: No expression;
- Score 1: Mild intensity expression;
- Score 2: Moderate intensity expression;
- Score 3: Intense expression.

Additionally, an analysis of the percentage of area immunostained for hBD-3 in each specimen was performed (quantitative analysis).^40^ The results were expressed as the mean of the percentages for the capsule and cystic epithelium in each specimen.

### Radiographic size measurement of periapical lesions

Radiographic images were analyzed using Image J 1.28 software for measurement of periapical radiolucent areas (in mm^2^) in both primary and permanent teeth, as previously described. Radiographic evaluations were performed using three calibrated examiners (Kappa > 0.8), and averaged. For calibration, the actual root size was measured and documented in the histopathological report.

### Evaluation of the relationship between the radiographic size of periapical lesions and percentage of hBD-3-immunostained areas

After the measurements, the periapical lesions were divided into the following groups:

- Small lesions (0–30 mm^2^);
- Large lesions (> 30 mm^2^).

The relationship between the radiographic size of the lesions (in mm^2^) and the percentage of area positive for hBD-3 expression in each specimen was evaluated in small and large lesions for both primary and permanent teeth.

### Statistical analysis

The distribution of the data was verified using the D’Agostino & Pearson normality test. Non-parametric data were compared using the Mann–Whitney test. Parametric data were compared using the *t*-test. Additionally, the Kruskal-Wallis test and Dunn's post-test were used for the analyses of inflammatory cells. Graph Pad Prism 9.0 software (Graph Pad Software Inc., San Diego, USA) was used for all the analyses. The significance level adopted was 5%.

## Results

### Analysis of the antimicrobial peptide (hBD-3) and its relationship with the radiographic lesion size

#### Characterization of the samples

Of the 11 cases of radicular cysts in the primary teeth with positive hBD-3 staining, 36.4% were from female subjects and 63.6% were from male subjects, with a mean age of 6.1 years. Furthermore, 72.7% of the radicular cysts in primary teeth were in the mandible and 27.3% were in the maxilla.

Of the 10 cases of cysts of permanent teeth with positive hBD-3 staining, 60% were from female subjects and 40% were from male subjects, with a mean age of 38.6 years. A total of 60% of the radicular cysts in permanent teeth were in the mandible and 40% were in the maxilla.

**Figure 1:**
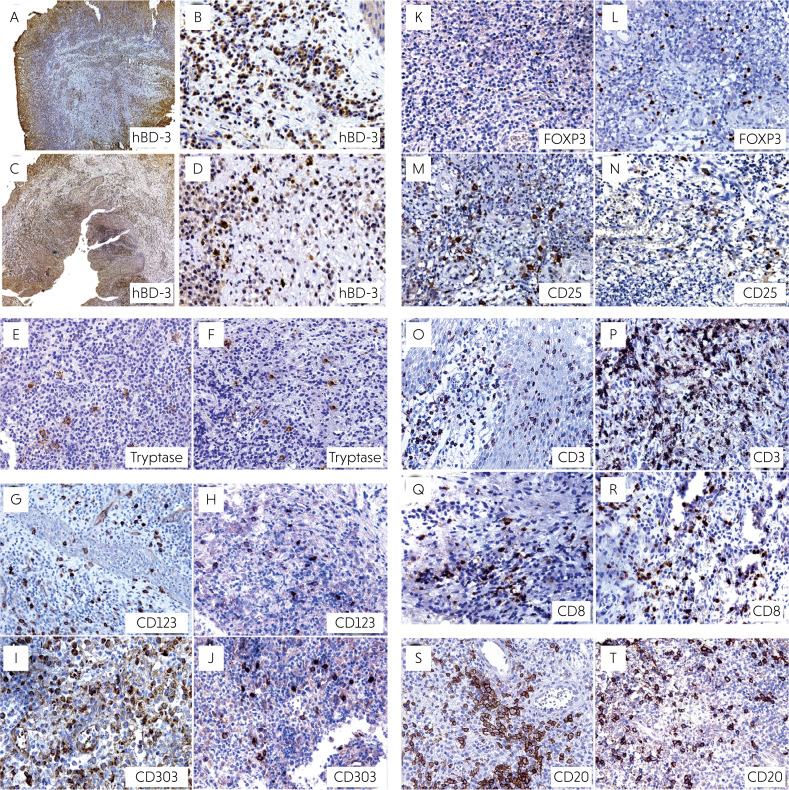
Representative photomicrographs showing the immunolabeling for hBD-3 (immunolabeled for hBD-3) in radicular cysts in primary (A,B) and permanent (C,D) teeth (Zeiss, ×5 and ×40), mast cells (immunolabeled for tryptase) in radicular cysts in primary (E) and permanent (F) teeth (Zeiss, ×40), plasmacytoid dendritic cells (immunolabeled for CD123) in radicular cysts in primary (G) and permanent (H) teeth, plasmacytoid dendritic cells (immunolabeled for CD303) in radicular cysts in primary (I) and permanent (J) teeth (Zeiss, ×40), Tregs (immunolabeled for FOXP3) in radicular cysts in primary (K) and permanent (L) teeth (Zeiss, ×40), Tregs (immunolabeled for CD25) in radicular cysts in primary (M) and permanent (N) teeth (Zeiss, ×40), lymphocytes (immunolabeled for CD3) in radicular cysts in primary (O) and permanent (P) teeth (Zeiss, ×40), cytotoxic T lymphocytes (immunolabeled for CD8) in radicular cysts in primary (Q) and permanent (R) teeth (Zeiss, ×40), B lymphocytes (immunolabeled for CD20) in radicular cysts in primary (S) and permanent (T) teeth (Zeiss, ×40).

Of the 14 analyzed cases of radicular cysts in primary teeth, hBD-3 staining was observed in 11 (78.5%) (Figures 1A and 1B). Similarly, among the 13 analyzed cases of cysts in permanent teeth, the staining was positive in 10 (76.9%) (Figure 1C and 1D).

#### Staining intensity scores in the immunohistochemical analysis

**Figure 2:**
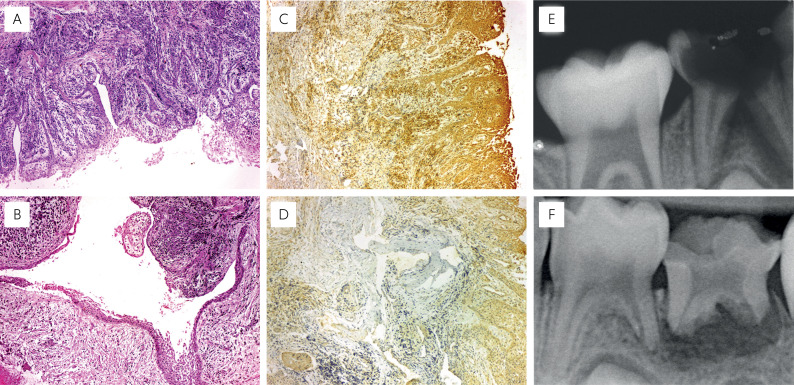
Staining intensity scores for hBD-3 in the epithelium and capsule after immunohistochemical evaluation of radicular cysts of primary (A) and permanent (B) teeth. Percentage of immunolabeled area for hBD-3 in small (0–30 mm2) and large (> 30 mm2) lesions after immunohistochemical and radiographic evaluation of radicular cysts in primary (C) and permanent (D) teeth. Different letters indicate a statistically significant difference (p < 0.05).

Table 2 presents the staining intensity scores for hBD-3 for the capsule and cystic epithelium. No statistically significant difference (p = 0.91) was found in the staining intensity scores for hBD-3 in the epithelium and cystic capsule in the cysts of primary teeth (Figure 2-A). Contrarily, the intensity was greater (p = 0.008) in the capsule than in the epithelium in permanent teeth (Figure 2-B).

**Table 2:** Distribution of the staining intensity scores for hBD-3 in the capsule and epithelium of Cysts from primary and permanent teeth.

Scores	Total	0		1		2		3	
Teeth	Cases	Capsule	Epithelium	Capsule	Epithelium	Capsule	Epithelium	Capsule	Epithelium
Primary	11	2	1	2	4	6	5	1	1
Permanent	10	0	2	2	6	8	2	0	0

### Evaluation of the relationship between the radiographic size of periapical lesions and percentage of regions immunostained for hBD-3

**Figure 3:**
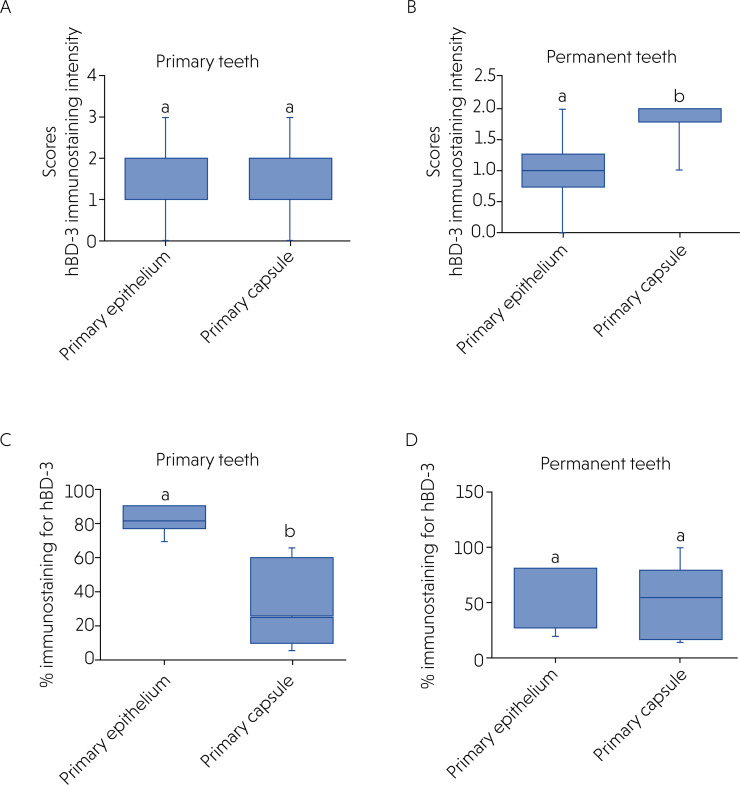
Representative photomicrographs showing hematoxylin and eosin (H&E) staining, indicative of a radicular cyst in primary teeth (A,B), Immunolabeling for hBD-3 in radicular cysts of primary teeth (C,D), with high expression immunolabeled for hBD-3 (C), and low expression immunolabeled for hBD-3 (D). Radiographs of periapical lesion in primary teeth (E,F). A higher immunolabeling for hBD-3 (C) was associated with a smaller size of the periapical lesion in primary teeth (E), whereas lower immunolabeling for hBD-3 (D) corresponded to a large lesion (F).

The data obtained for primary and permanent teeth were expressed radiographically in terms of area (mm^2^) and using immunohistochemistry in terms of the percentage of the region immunostained for hBD-3 (Table 3, Figure 3).

**Table 3:** Distribution of periapical lesion areas (mm^2^) and percentages of regions immunostained for hBD-3 after radiographic and immunohistochemical evaluation of radicular cysts in primary (A) and permanent (B) teeth.

Cases primary	Area (mm^2^)	Percentages of regions immunostained for hBD-3 (%)	Cases ppermanent teeth	Area (mm^2)^	Percentages of regions immunostained for hBD-3 (%)
Teeth					
1	7,9	85	1	18,2	80
2	9,4	70	2	19,2	80
3	12,7	90	3	8,9	35
4	22,9	90	4	27,8	20
5	29,1	80	5	27,8	65
6	33,4	80	6	48	100
7	42,1	15	7	80	55
8	45,5	65	8	150,3	15
9	58,4	25	9	160	20
10	65,5	55	10	358,6	58
11	184,8	5			

In primary teeth, small lesions had a median of 82.50 (Q1 = 77.50; Q3 = 90.00) immunostained cells, whereas this value was 25.00 (Q1 = 10.00; Q3 = 60.00) in large lesions; the difference between the two was statistically significant (p = 0.0078) (Figure 2-C).

In permanent teeth, small lesions had a median of 65.00 (Q1 = 27.50; Q3 = 80.00) immunostained cells, whereas this value was 55.00 (Q1 = 17.50; Q3 = 78.75) in large lesions; the difference between the two was not statistically significant (p = 0.599) (Figure 2-D).

### Quantification of the different immune and inflammatory cell types

#### Characterization of the sample

Of the 19 analyzed cases of radicular cysts in primary teeth, 36.8% were from female subjects and 63.2% were from male subjects, with a mean age of 6.3 years. Among these, 63.2% of the radicular cysts in primary teeth were located in the mandible and 36.8% were in the maxilla.

Of the 17 cases of permanent tooth cysts, 58.8% were female and 41.2% were male, with a mean age of 51.4 years. A total of 70.6% of the radicular cysts in permanent teeth were in the mandible and 29.4% were in the maxilla.

### Immunohistochemical analysis

**Figure 4:**
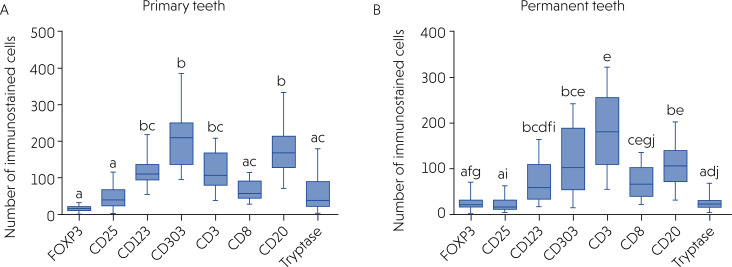
Comparison of the number of cells immunolabeled for tryptase, FOXP3, CD25, CD123, CD303, CD3, CD8, and CD20 in radicular cysts in primary (A) and permanent (B) teeth using Kruskal–Wallis test and Dunn's post-test. Different letters indicate statistically significant difference between groups (p < 0.05).

The immunohistochemical analysis showed immunostaining for tryptase (mast cells), CD123 and CD303 (plasmacytoid dendritic cells), FOXP3 and CD25 (Tregs), CD3 (T lymphocytes), CD8 (cytotoxic T lymphocytes), and CD20 (B lymphocytes) in all cases (100%), in primary and permanent teeth. The most prevalent cells in the radicular cysts in primary teeth were mature (CD123+) and immature (CD303+) plasmacytoid dendritic cells, T lymphocytes (CD3+), and B lymphocytes (CD20+) (p < 0.05). In contrast, in permanent teeth, the most prevalent cells were T lymphocytes (CD3+), immature plasmacytoid dendritic cells (CD303+), cytotoxic T lymphocytes (CD8+), and B lymphocytes (CD20+) (*p* < 0.05). Figure 4 shows a comparison of cell quantities using the Kruskal-Wallis test and Dunn's post-test for the evaluated markers.

### Mast cells

**Figure 5:**
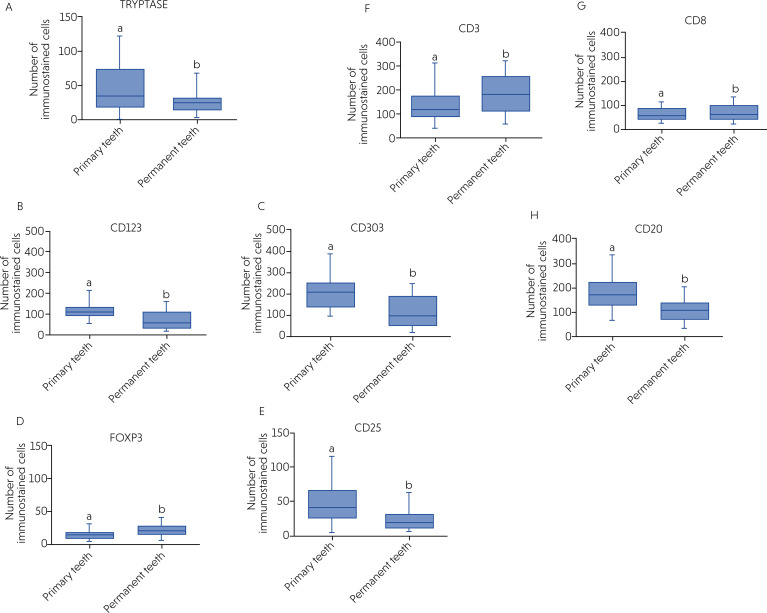
Comparison of the number of mast cells (immunolabeled for tryptase) (A), plasmacytoid dendritic cells [cells immunolabeled for CD123 (B) and CD303 (C)], Treg [cells immunolabeled for FOXP3 (D) and CD25 (E)], lymphocytes [cells immunolabeled for CD3 (F)], cytotoxic T lymphocytes [cells immunolabeled for CD8 (G)], and B lymphocytes [cells immunolabeled for CD20 (H)] in the radicular cysts in primary and permanent teeth. The same letters indicate the absence of a statistically significant difference (p < 0.05).

A comparison of the number of cells in the radicular cysts immunostained for mast cell marker indicated that primary teeth had a median of 34.15 cells (Q1 = 18.14; Q3 = 73.89) (Figure 1-E) whereas permanent teeth had a median of 22.65 cells (Q1 = 14.63; Q3 = 31.15) (Figure 1-F). Despite the numerical difference between the groups, no statistically significant difference was found between the number of mast cells in the radicular cysts in primary and permanent teeth (p = 0.119) (Figure 5-A).

No statistically significant difference was found in the number of non-degranulated mast cells (Q1 = 15; Q3 = 37.5) and degranulated mast cells (Q1 = 3.750; Q3 = 45.25) in the primary teeth (p = 4.812). In contrast, in permanent teeth, when comparing non-degranulated mast cells (Q1 = 10; Q3 = 17.25) and degranulated mast cells (Q1 = 0; Q3 = 11), a statistically significant difference was observed between the cell types, with a significantly higher number of non-degranulated mast cells than degranulated mast cells (p = 0.0082).

After comparing the number of non-degranulated mast cells in primary and permanent teeth, a significant difference was found between primary (Q1 = 15; Q3 = 37.5) and permanent teeth (Q1 = 10; Q3 = 17.25), with a significantly higher number in primary teeth (p = 0.0211). Additionally, when comparing non-degranulated mast cells in primary (Q1 = 3.750; Q3 = 45.25) and permanent teeth (Q1 = 0; Q3 = 11.00); although there was a numerical trend toward higher values in the primary teeth, no statistically significant difference was observed between the groups (p = 0.0513).

## Plasmacytoid dendritic cells

The median number of mature plasmacytoid dendritic cells, as evidenced by staining for the CD123 marker, was 110.8 (Q1 = 94.0; Q3 = 135.8) in primary teeth (Figure 1-G) and 57.0 (Q1 = 32.38; Q3 = 107.2) in permanent teeth (Figure 1-H). The difference between these two groups was statistically significant (p = 0.008). Thus, radicular cysts from primary teeth showed significantly higher numbers of mature plasmacytoid dendritic cells than those from the permanent teeth (Figure 5-B).

The number of plasmacytoid dendritic cells was also evaluated by analyzing the CD303 marker (which is a marker of immature dendritic cells). The mean number of immunostained cells was 208.6 (dp = 84.63) in cysts from primary teeth (Figure 1-I), whereas it was 115.4 (dp = 72.12) in cysts from permanent teeth (Figure 1-J). Thus, the mean number of immature plasmacytoid dendritic cells in the radicular cysts in primary teeth was also significantly higher than that in permanent teeth (p = 0.001) (Figure 5-C).

### Regulatory t lymphocytes

The average number of mature Tregs evaluated using the FOXP3 marker, was 14.88 (dp = 6.58) in the cysts of primary teeth (Figure 1-K) and 20.56 (dp = 9.12) in those in permanent teeth (Figure 1-L). A statistical analysis of these data showed that the number of mature Tregs in the cysts in permanent teeth was significantly higher than that in the cysts in primary teeth (*p* = 0.04) (Figure 5-D).

The average number of immature Tregs evaluated using the CD25 marker, was 45.54 (dp = 27.27) in the cysts in primary teeth (Figure 1-M) and 22.26 (dp = 17.75) in those in permanent teeth (Figure 1-N). The average number of immature Tregs in the cysts in primary teeth was statistically higher than that in the cysts in permanent teeth (*p* = 0.011) (Figure 5-E).

### T lymphocytes

The median number of T lymphocytes, evaluated using the CD3 marker, was 114.0 (Q1 = 86.0; Q3 = 172.8) in primary teeth (Figure 1-O) and 181.3 (Q1 = 110.0; Q3 = 256.8) in those in permanent teeth (Figure 1-P). Thus, the median number of T lymphocytes in the cysts in permanent teeth was significantly higher (p = 0.044) than that in primary teeth (Figure 5-F).

### Cytotoxic T lymphocytes

The mean number of cytotoxic T lymphocytes, evaluated using the CD8 marker, was 61.82 (dp = 24.47) in primary teeth (Figure 1-Q) and 70.29 (dp = 36.08) in those in permanent teeth (Figure 1-R). No statistically significant difference was evident between primary and permanent teeth (*p* = 0.411) (Figure 5-G).

### B lymphocytes

The mean number of B lymphocytes, evaluated using the CD20 marker, was 172.0 (dp = 75.68) in primary teeth (Figure 1-S) and 106.3 (dp = 44.34) in the cysts in permanent teeth (Figure 1-T). A statistical analysis demonstrated that the number of B lymphocytes in primary teeth was significantly higher than that in the cysts in permanent teeth (p = 0.0024) (Figure 5-H).

## Discussion

Harder et al.^41^ have suggested that hBD-3 might play an important role in the defense against microorganisms, especially in the epithelium. The presence of hBD-3 has been reported in the oral mucosal epithelium, airway epithelium, corneal epithelium, nasal and urogenital mucosal epithelium, endothelial hair cell epithelium, intestinal tissue epithelium, prostate epithelium and gingival epithelium.^18^ However, the present study is the first to demonstrate the expression of hBD-3 in the epithelium and capsule of radicular cysts of primary and permanent teeth. Karaka et al. observed that epithelial cells exposed to periodontopathogenic microorganisms expressed hBD-3. The findings of the present study are in agreement with this because we demonstrated that the endodontic infection process stimulates hBD-3 production process stimulates hBD-3 production.

In the present study, the percentage of cases expressing hBD-3 was similar in radicular cysts in primary and permanent teeth; in primary teeth, the expression was the same in the capsule and epithelium, whereas in permanent teeth, it was greater in the capsule, which was related to the presence of immune system cells such as macrophages, lymphocytes, and plasma cells.

Yu et al.^43^ evaluated the role of hBD-3 in the modulation and activation of macrophages in animals during a lipopolysaccharide-induced acute inflammatory response. They concluded that hBD-3 modulates the differentiation of macrophages into the M2 phenotype, contributing to their anti-inflammatory role. With respect to lymphocytes, hBD-3 may provide signals for the recruitment of T and dendritic cells to the site of infection. Plasma cells are also involved in releasing hBD-3 upon infection.^44^

An important finding of this study was the higher expression of hBD-3 in small lesions (less than 30 mm^2^) than that in large lesions (greater than 30 mm^2^). This indicates that the expression of hBD-3 in the epithelium/capsule of radicular cysts in primary teeth may act as a protective mechanism employed by organisms to circumscribe and limit the lesion. These results highlight the important protective role of endogenous hBD-3 in periapical lesions of primary teeth. In contrast, no difference in hBD-3 expression was observed between small and large lesions in permanent teeth, which could possibly be due to other factors, including the production of other AMPs, other defensins, histatins, and cathelicidins. Factors such as the patient's inherent resistance^45^ and the evolutionary stage of the lesion^46^ may influence the higher or lower expression of hBD-3 and could act as confounding factors in the obtained results. Therefore, in the present study, only patients with good overall health, as determined by a thorough medical history, were selected, encompassing lesions at different stages of development (small and large lesions). However, further studies are required in this regard.

Another aspect evaluated in the present study was the different cell types (non-degranulated and degranulated mast cells, plasmacytoid dendritic cells, Tregs, T lymphocytes, cytotoxic T lymphocytes, and B lymphocytes) that were identified in 100% of the radicular cysts of primary and permanent teeth. As pointed out by Teixeira et al.^16^, these cells produce a complex environment with high cytokine production, which is involved in the etiology of periapical lesions.

However, few immunohistochemical studies have evaluated periapical lesions in primary human teeth. Bolan et al.^11^ evaluated the presence of T and B lymphocytes and macrophages in periapical lesions, general granulomas, epithelialized granulomas, acute abscesses, and chronic abscesses of primary teeth and concluded that humoral and cell-mediated immune responses are important in these lesions. In a recent study by our research group^12^ evaluating the polarization of M1 and M2 macrophages in radicular cysts of human primary and permanent teeth using immunohistochemistry, no significant differences were observed. This is extremely relevant considering the immaturity of the immune system in children and the potential local and systemic effects of infections and triggered reactions.^13^

Dendritic cells are components of the innate immune system, performing important functions in cell-mediated immune reactions as well as in the pathogenesis of periapical lesions.^16^ These cells are responsible for antigen presentation to T cells and are extremely important in the release of inflammatory mediators, which activate Th1, Th2, and Th17 cells or Tregs.^41^ In the present study, we identified plasmacytoid dendritic cells in 100% of the cases of radicular cysts in primary and permanent teeth. Immature plasmacytoid dendritic cells were the most prevalent cell type in the primary dentition and the second most prevalent cell type in the permanent dentition.

Weber et al.^5^ evaluated antigen-presenting cells (dendritic cells and macrophages) and T lymphocytes in radicular cysts of permanent teeth and concluded that these cells are relevant to the pathophysiology of periapical injury. However, their study differed from the present study in that it evaluated mature myeloid dendritic cells using the CD83 marker.

It should be emphasized that there have been no published studies on the evaluation of plasmacytoid dendritic cells in periapical lesions in primary teeth; therefore, it is impossible to provide a comparative perspective of our results. Additionally, in the present study, we detected, for the first time, the presence of immunostained plasmacytoid dendritic cells (mature and immature) in radicular cysts in primary teeth in larger numbers than that in permanent teeth (*p* < 0.05).

Similar to dendritic cells, mast cells are also a component of the innate immune system and are considered protective cells.^8, 48^ In comparison to dendritic cells, in this study, mast cells were found in smaller numbers, although they were present in 100% of the cases of primary and permanent teeth, with no significant difference between them (p > 0.05).

Although mast cells have not been evaluated in periapical lesions of primary teeth in the literature, some researchers have evaluated these cells in granulomas and radicular cysts in permanent teeth.^8, 9^ Shiromany et al.^8^ reported the presence of mast cells in cysts in the connective tissue immediately below the epithelium, as was also observed in the present study, both in radicular cysts in primary and permanent teeth.

The present study demonstrated immunopositivity for mast cells (non-degranulated and degranulated) in all cases. This finding confirms the statement by Kamboj et al.^47^ that mast cells play a crucial role in the pathogenesis of periapical lesions, driving initiation, progression, chronicity, and fibrosis, and potentially serve as markers of disease activity. A novel finding of the present study is that radicular cysts of primary teeth exhibited higher numbers of non-degranulated mast cells and lower numbers of degranulated mast cells (p < 0.05) when compared to permanent teeth, which may be attributed to the less active participation of mast cells in the pathological process of primary radicular cysts. Another cell type of adaptive immunity evaluated in this study was T lymphocytes; these were present in 100% of the cases of radicular cysts in primary and permanent teeth but were more abundant in the permanent dentition (p < 0.05). Based on this finding, we inferred that the late response mediated by T lymphocytes occurs more effectively in adults. Tregs and cytotoxic T lymphocytes were also evaluated. Tregs play an important role in the control of the inflammatory microenvironment in radicular cysts^48^ and express IL-10 and TGF-β in periapical lesions in permanent teeth.^7^ We identified mature and immature Tregs using FOXP3 and CD25 immunostaining^48^ in 100% of the cases of radicular cysts in primary and permanent teeth.

We also verified a higher expression of mature Tregs in radicular cysts in permanent teeth and a higher expression of immature Tregs in radicular cysts in primary teeth. Thus, it can be hypothesized that in radicular cysts in primary teeth, that is, in children, the maturation of Tregs does not occur as effectively as in adults. According to Zhang, Guo, and Jia,^49^ Tregs contribute to the homeostasis of the host immune response and minimize tissue damage in periapical lesions. Thus, the study of these cells may be useful for the future implementation of immunotherapy for the treatment of these lesions.

According to Stashenko and Yu,^50^ cytotoxic T lymphocytes are associated with the chronic phase of lesions. In the present study, we demonstrate that these cells were present in radicular cysts in primary teeth, with no significant difference (*p* > 0.05) in their numbers compared to those in permanent teeth, although there is a tendency for higher concentrations of CD8+ T lymphocytes in radicular cysts in permanent teeth.

B lymphocytes produce antibodies and are components of humoral immunity Polanco et al.^3^ characterized the immunoglobulins (light and heavy chain) present in granulomas and cysts and reported significant difference between cysts and periapical granulomas of primary and permanent teeth, with higher IgG expression in radicular cysts in primary teeth.

In the present study, unlike for T lymphocytes, the number of B lymphocytes in radicular cysts in primary teeth was greater than that in permanent dentition (p < 0.05), which can be explained by the fact that although B lymphocytes are classified as cells of adaptive immunity, they also have roles in innate immunity, probably representing a primitive and conserved form of immunity.^3^

The findings of the present study provide a better comparative understanding of pulpal and periapical pathology in primary and permanent teeth, which would be useful for devising targeted treatment techniques and immunologically active materials for endodontic use, as has also been suggested by Weber et al.^5^, Braz-Silva et al.^14^, Zhang et al.^49^, Wang et al.^15^ and Teixeira et al.^16^

These results may also open new avenues, such as the development and production of materials that stimulate the expression of endogenous hBDs or employ recombinant hBDs, contributing to the success of endodontic treatment.

## Data Availability

The authors declare that all data generated or analyzed during this study are included in this published article.
